# Echocardiographic Evaluation of Pericardial Effusion and Cardiac Tamponade

**DOI:** 10.3389/fped.2017.00079

**Published:** 2017-04-24

**Authors:** Alejandro Pérez-Casares, Sergi Cesar, Laia Brunet-Garcia, Joan Sanchez-de-Toledo

**Affiliations:** ^1^Department of Pediatric Cardiology, Hospital Sant Joan de Déu Barcelona, University of Barcelona, Barcelona, Spain; ^2^Division of Cardiac Intensive Care, Children’s Hospital of Pittsburgh, University of Pittsburgh, Pittsburgh, PA, USA

**Keywords:** pericardial effusion, cardiac tamponade, echocardiography, pericardium, ultrasound

## Abstract

Pericardial effusion (PEff) is defined by an increase in the physiological amount of fluid within the pericardial space. It can appear following different medical conditions, mainly related to inflammation and cardiac surgery. Cardiac tamponade is a critical condition that occurs after sudden and/or excessive accumulation of fluid in the pericardial space that restricts appropriate filling of the cardiac chambers disturbing normal hemodynamics and ultimately causing hypotension and cardiac arrest. It is, therefore, a life-threatening condition that must be diagnosed as soon as possible for correct treatment and management. Echocardiographic evaluation of PEff is paramount for timely and appropriate diagnosis and management. A structured echocardiographic approach including two-dimensional, M-mode, and Doppler echocardiographic evaluation assessing (i) quantity and quality of pericardial fluid, (ii) collapse of cardiac chambers, (iii) respiratory variation of the ventricular diameters, (iv) inferior vena cava collapsibility, and (v) flow patterns in atrioventricular valves should give the bedside clinician the necessary information to appropriately manage PEff. Here, we review these key echocardiographic signs that will ensure an appropriate assessment of a patient with PEff and/or cardiac tamponade.

## Introduction

Diagnosis of pericardial conditions can be difficult to reach when relying solely on physical examination and clinical findings. Signs and symptoms of significant pericardial effusion (PEff) include tachycardia, raised jugular venous pressure, orthopnea, *pulsus paradoxus* (drop of blood pressure of at least 10% during inspiration), and pericardial rub (only in case of pericarditis). Hypotension and bradycardia will appear ultimately before cardiac arrest.

The use of ultrasound in the diagnosis and evaluation of pericardial disease was initially described in 1955 by Edler (1). Since then, it has remained the gold standard imaging technique. Ultrasound has the advantage of being a rapid, innocuous, and readily available technique at the patient’s bedside (1–6). Recently, the use of cardiovascular magnetic resonance (CMR) and computed tomography (CT) has also helped in the diagnosis and management of pericardial disease (7). Nevertheless, echocardiography still remains the imaging modality of choice to assess the pericardium due to its ease of use, availability, cost-effectiveness and its comprehensive appraisal of the heart and its hemodynamics.

## Anatomy of the Pericardium

The pericardium constricts the heart into position, limiting distension of the cardiac chambers and also protecting it from infections within the chest. The outermost layer of the pericardium is formed by a fibrous envelope composed primarily of collagen fibers with interspersed short elastic fibrils (8). The fibrous envelope is continuous to the adventia of the great vessels superiorly and attached to the central tendon of the diaphragm inferiorly. It is attached anteriorly to the sternum by sternopericardial ligaments. Posteriorly, it is anatomically related to the bronchi, esophagus, and the thoracic descending aorta. The inner layer of the pericardium is composed of visceral and parietal layers. The space between these two inner layers is the pericardial cavity and normally contains a small amount of fluid (<50 ml, which serves as lubricant between the visceral and the parietal pericardial layers) (9). The visceral layer covers the epicardium, where a variable amount of fat is found. Parietal layer has a variable thickness (0.7–1.7 mm) as described from echocardiogram, CT, and CMR (10–12). For this reason, the use of transthoracic echocardiography may not be optimal, due to hyperechogenic signal received from epicardial fat.

### Absence of the Pericardium

**Figure 1:**
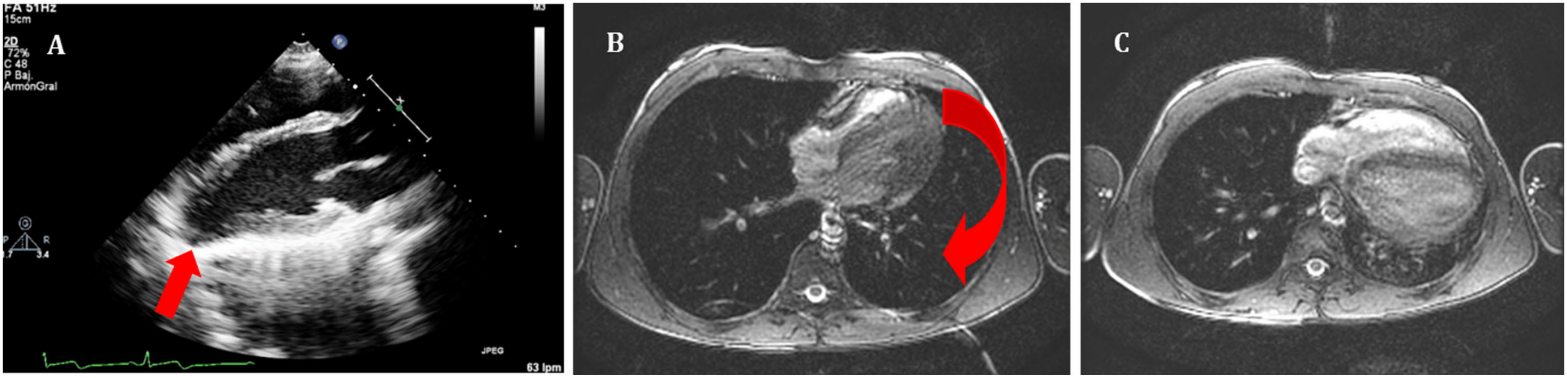
**(A)** Echocardiography in parasternal long axis. Arrow shows the posterior displacement of the apex and the lack of the typical round left ventricular apex. Cardiovascular magnetic resonance showing the clockwise rotation. Compared with the prone position **(B)**, leftward posterior rotation of the heart is seen in when patient is in supine position **(C)**.

Absence of the pericardium is a rare condition due to failure in the embryogenesis of the pericardial sac. This anomaly can be classified based on extension (total or partial) and location (left, right, or bilateral) (13). Complete absence of left pericardium sac is most common (9); however, patients are usually asymptomatic and diagnosis often incidental even though symptoms such as chest pain, palpitations, or dyspnea have been described. Partial absence of the pericardium can lead to sudden death due to compression, herniation, or cardiac strangulation (14). Chest radiography, electrocardiogram, echocardiography, CT, and CMR have proven helpful diagnostic studies. Specifically, echocardiography is useful, since the image of the parasternal long-axis view showing a posterior and leftward displacement of the apex without its normal round shape is different than the usual one (Figure 1). No intervention is needed if patients are asymptomatic, but surgery is indicated in symptomatic cases or in those at high risk of herniation (partial defects) (15).

## Pericardial Effusion

### Physiology

**Figure 2:**
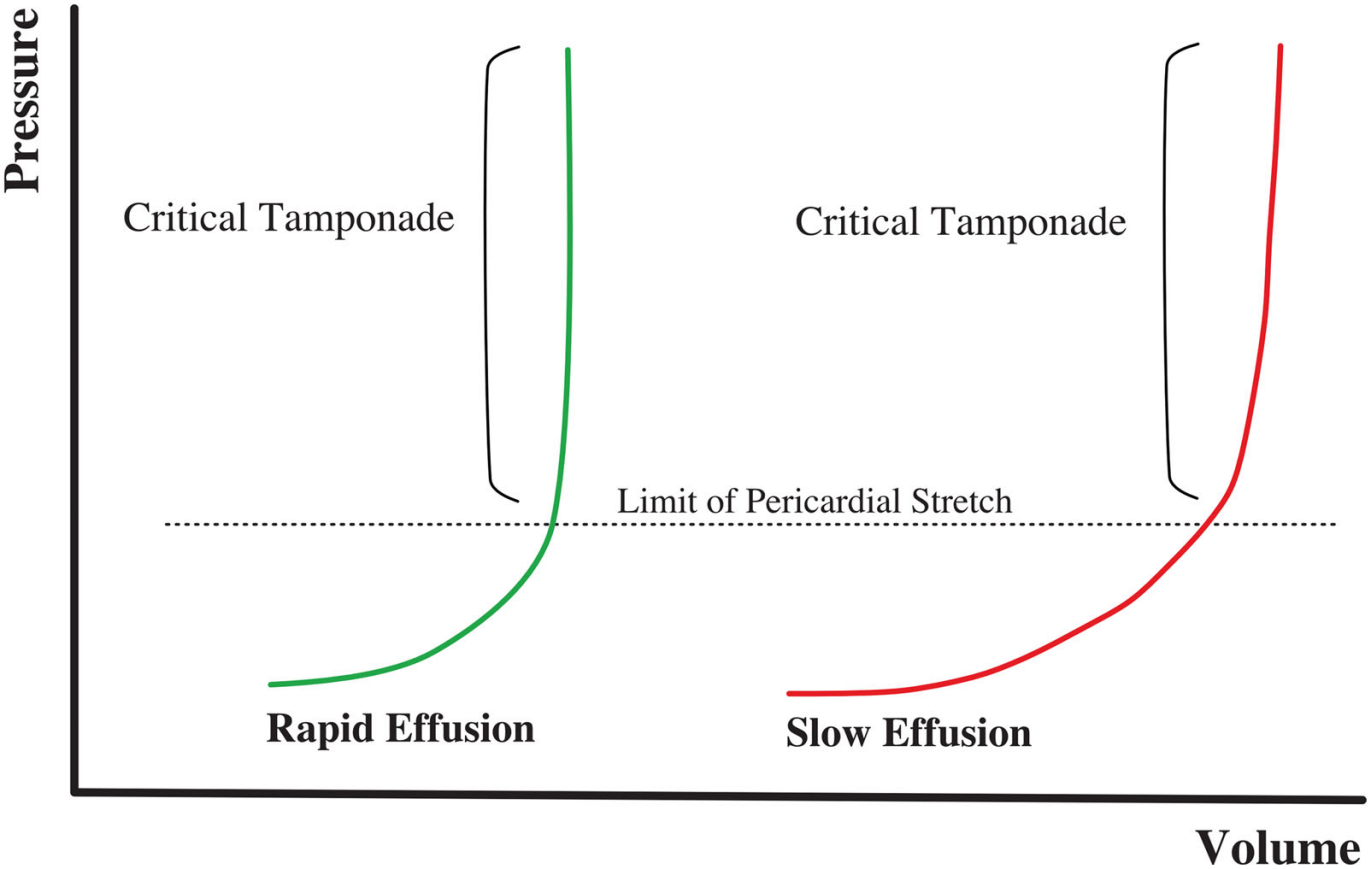
**Volume–pressure relationship in cardiac tamponade physiology**. Rapid onset effusion leads faster to cardiac tamponade, comparing to a slow increase of the pericardial fluid that will allow the pericardium to stretch reaching the critical point with a higher volume of effusion.

Pericardial effusion is defined by an increase in the amount of fluid within the pericardial space. Etiology is very diverse. Tuberculosis is the primary cause in developing countries, while viral infections and postsurgical complications are the most frequent causes in developed countries (16–18). Parietal pericardium compliance follows a non-linear relationship (Figure 2). Therefore, the effect on the cardiac chambers and resulting clinical symptoms from PEffs will not only depend on the etiology but also on the amount of fluid accumulation and the time since onset.

There is an initial flat part of the curve, when the increase of pericardial fluid represents either no or minimum pressure increases within the pericardial space. However, once a threshold point is reached, small changes in fluid volume can cause large pressure variations in the intrapericardial space. Thus, an acute onset, small PEff can cause cardiac tamponade, while chronic and slow PEff may allow the elastic fibrils of the pericardium to stretch so that the volume–pressure curve is shifted to the right, causing minimal effects on the hemodynamics (19).

### Echocardiography

Echocardiography should be the initial imaging modality used should PEff be suspected (7, 20). It is an innocuous technique, highly reliable, and readily available at the patient’s bedside. Due to its versatility, echocardiography is the technique of choice to identify hemodynamic compromise in the setting of PEff.

**Figure 3:**
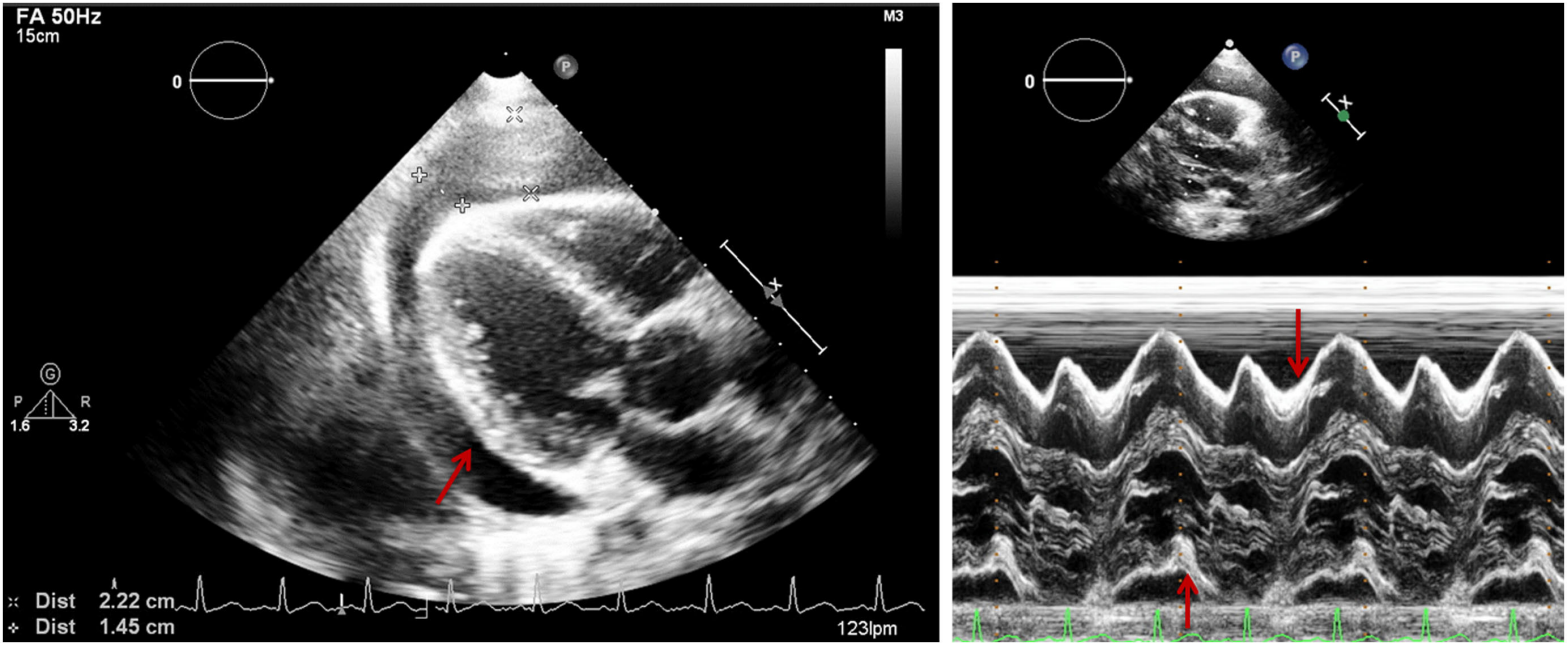
**Parasternal long axis (two-dimensional on the left and M-mode on the right) in a patient with pericardial effusion (PEff)**. Arrow shows echo-free signal from pericardial fluid. M-mode shows PEff only during systole.

**Figure 4:**
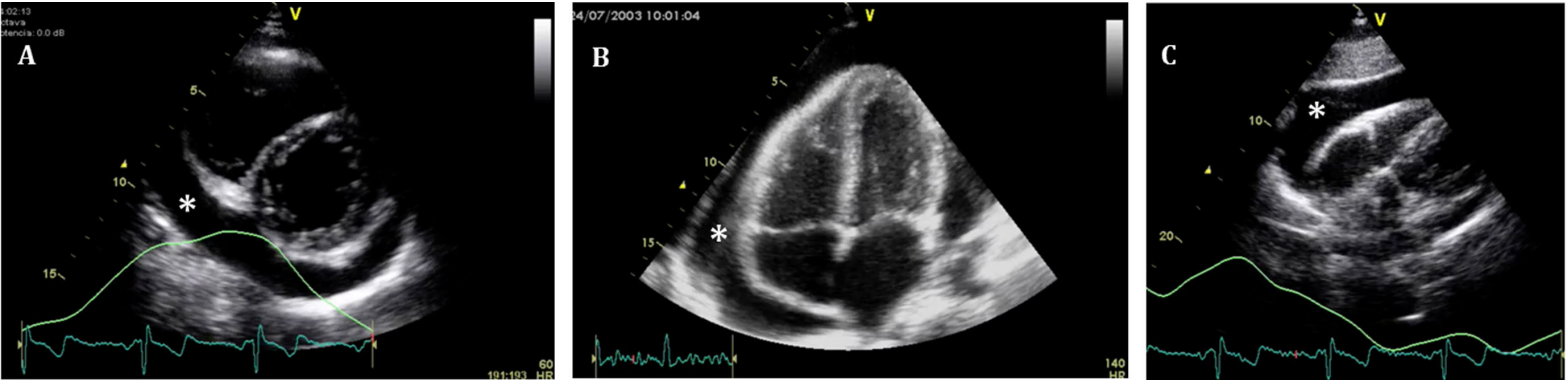
Standard echocardiographic views to assess a pericardial effusion (*). **(A)** Parasternal short axis; **(B)** four-chamber view; and **(C)** subcostal view.

Pericardial effusion appears as an echo-free space between the two layers of the pericardium. Echocardiographic assessment should be structured and focused to (i) differentiate between global or localized effusion; (ii) quantify the effusion; (iii) describe fluid appearance; and (iv) analyze hemodynamic compromise. Standard views along with two-dimensional (2D) echocardiographic, M-Mode, and Doppler analysis should be routinely used in the assessment of PEff. Four standard views should be used for reproducibility [subcostal, four-chamber, and parasternal long and short axis (Figures 3 and 4)]; however, in emergent situations, a single view (usually the subcostal one) is enough to diagnose significant PEff. A combination of the aforementioned four standard views should be used to determine whether the effusion is global or localized.

Quantitative assessment of the PEff can be helpful for diagnosis and follow-up. However, as previously mentioned, the volume of PEff does not necessarily correlate with the clinical symptoms. PEffs in adults are commonly classified according to the size including trivial (seen only in systole), mild (<10 mm), moderate (10–20 mm), and severe (>20 mm) (21). The most common and easy view to reproduce is the parasternal long axis (Figure 3), but it is still important to compare with other standard views (Figure 4), such as the parasternal short axis and the four-chamber view. A subcostal view is also very commonly used, but it is important to know that depending on the angle of the probe, it is easy to overestimate the size of the effusion. At end diastole, echo-free space between both pericardial layers can be measured along the posterior wall of the left ventricle (LV). M-mode echocardiography in the parasternal long axis shows an echo-free space throughout between the visceral and the parietal pericardium. This sign is only seen in systole in case of mild effusions and during the complete cycle when the effusion is at least moderate.

Since PEff can be of different etiologies (7), it is usually difficult to make an appropriate qualitative assessment of fluid characteristics just by echocardiographic signal. A qualitative assessment can be made by defining whether it is global or loculated, transudative, exudative, or containing clotted blood or fibrin. Intrapericardial fibrin and pyogenic exudates can create tabications and loculate the effusion. Particular echogenic densities such as the ones seen in complex exudates or hemopericardium with the presence of fibrin strands and clots are easily recognized by echocardiography. On the other hand, transudates will usually show a more echo-free signal giving a characteristic sinusoid image in M-mode echocardiography that could help differentiate complex exudates from transudates. Hemopericardium can be differentiated because of the spontaneous contrast signal resulting from the swirling of the blood within the pericardial space (Figure 6). Nevertheless, a complete clinical assessment complemented by laboratory tests and imaging studies should always be taken into consideration to find the underlying condition (22).

## Cardiac Tamponade

### Physiology

**Figure 5:**
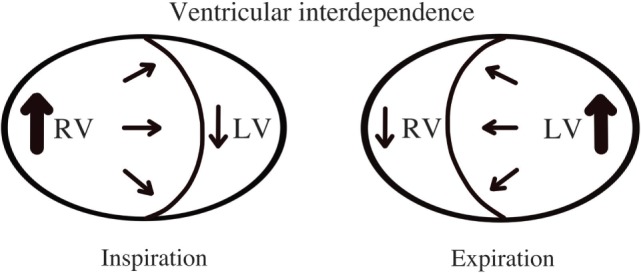
**Ventricular interdependence**. Diagram shows physiological hemodynamic changes in the cardiac chambers within the respiratory cycle.

Cardiac tamponade is a life-threatening condition caused by accumulation of fluid in the pericardial space that compresses the cardiac chambers and restricts them from normal filling (9, 23, 24). The rate of fluid accumulation will determine the clinical manifestations. As the pericardial space is filled with fluid, increased pressure within the pericardial space will overpower the relaxing pressures of the cardiac chambers, which varies during the respiratory cycle. During spontaneous inspiration, regardless of presence of cardiac tamponade, systemic venous return is increased and filling pressures of the right ventricle (RV) are higher than the filling pressures of LV (Figure 5) (25). If left ventricular diastolic filling pressures are equal or lower than intrapericardial pressures, the volume the ventricle can accept will be restricted, causing a decrease in the cardiac output. An exaggerated inspiratory drop in the systemic blood pressure (>10 mmHg) is known as *pulsus paradoxus* and is a typical sign of significant PEff (sensitivity of 80% and specificity of 40%) (26).

**Figure 6:**
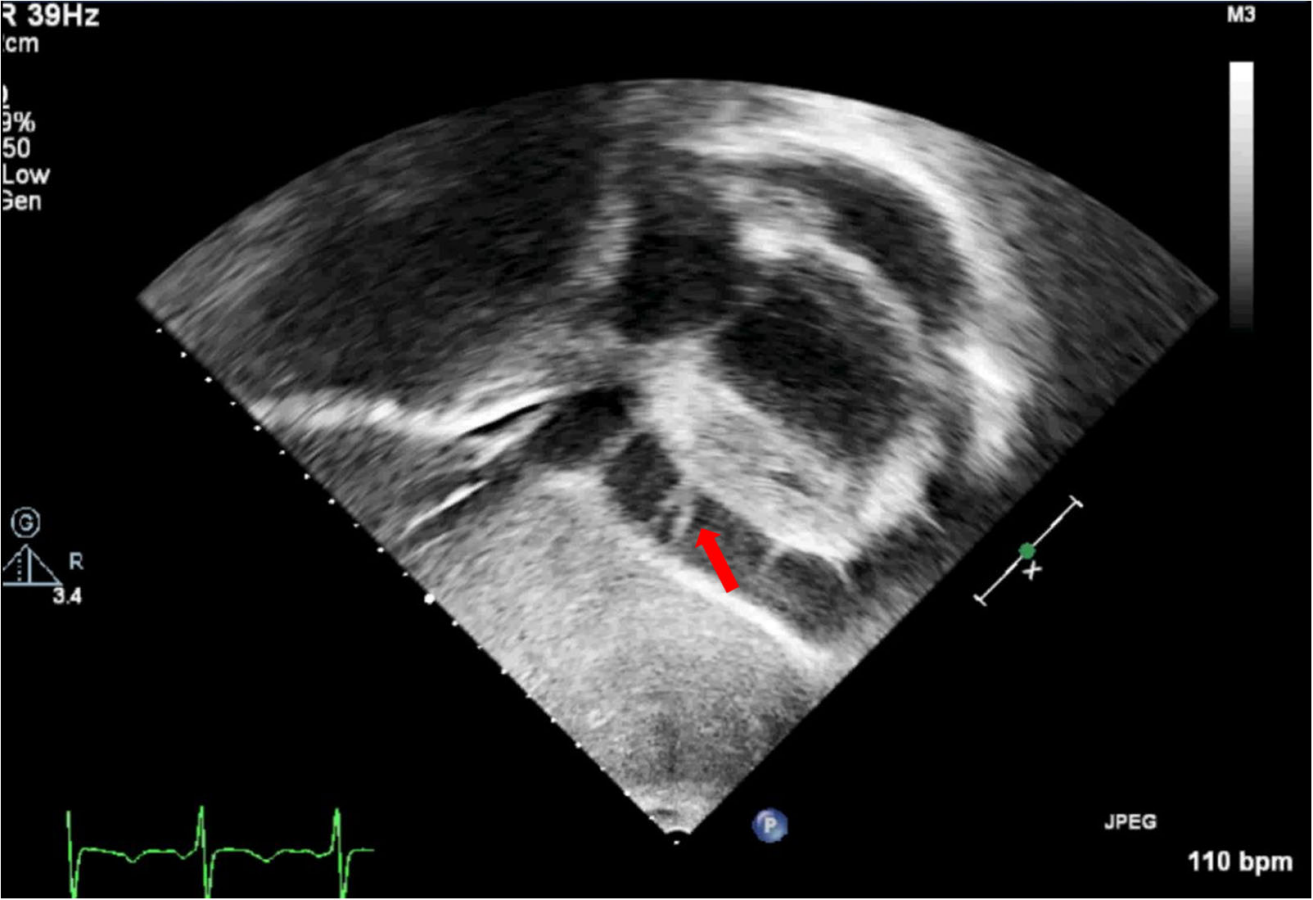
**Subcostal view showing fibrin strains in a large pericardial effusion**.

As cardiac tamponade becomes more severe, compensatory mechanisms will ultimately fail, preload will be insufficient to sustain cardiac filling, coronary perfusion will be in jeopardy leading to rapid bradycardia and abrupt blood pressure drop as the usual terminal event.

### Echocardiography

Echocardiography should be obtained immediately if cardiac tamponade is suspected. The information obtained from this study should assess (i) quantity and quality of pericardial fluid; (ii) collapsibility of cardiac chambers; (iii) diastolic ventricular size variability with respiratory cycle; (iv) septal “bounce”; (v) collapsibility of the inferior vena cava (IVC); respiratory variation of flow patterns through and semilunar valves; and (vi) hepatic and pulmonary veins flow patterns. The first five signs are easily obtained with both 2D echocardiography and M-mode. Assessment of flow pattern variability will require Doppler evaluation.

#### 2D Echocardiography and M-Mode

**Figure 7:**
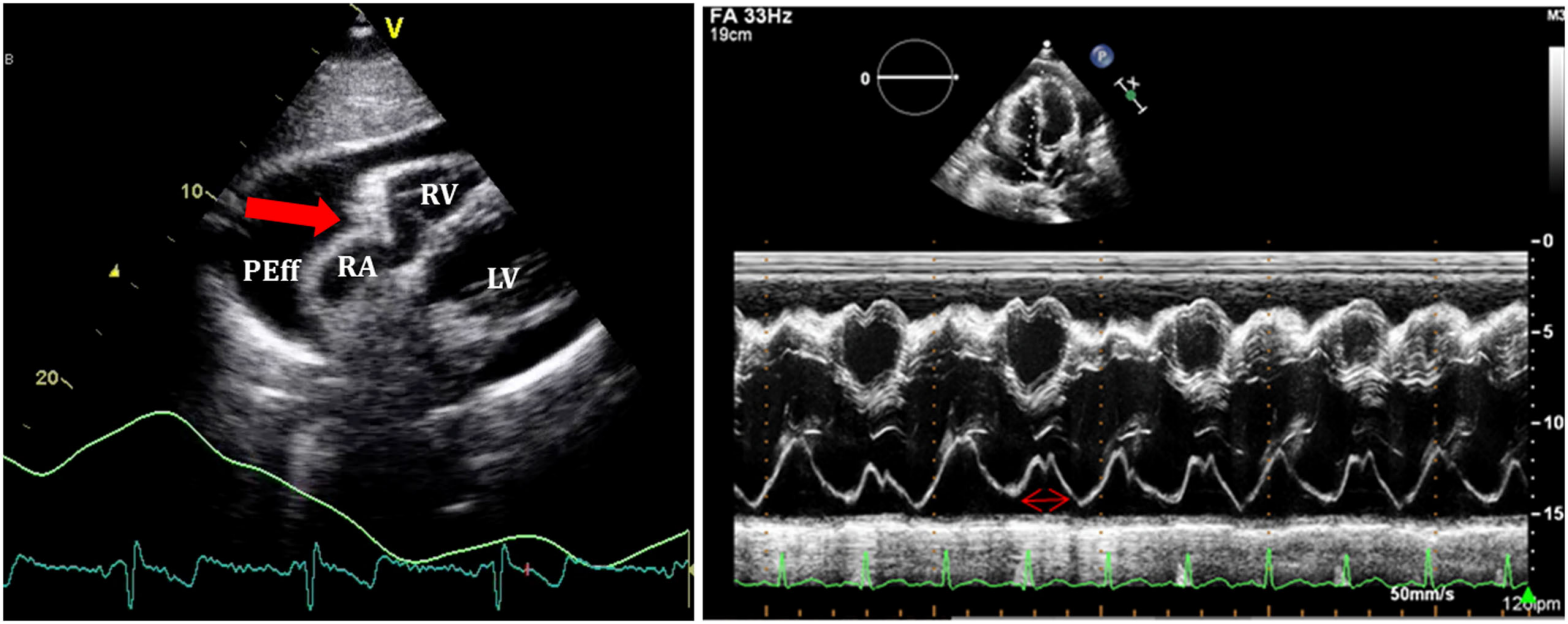
**On the left, subcostal view of a patient with significant pericardial effusion and evidence of right atrium (RA) collapse**. On the right, M-mode through RA shows collapse duration over 1/3 of the systole.

**Figure 8:**
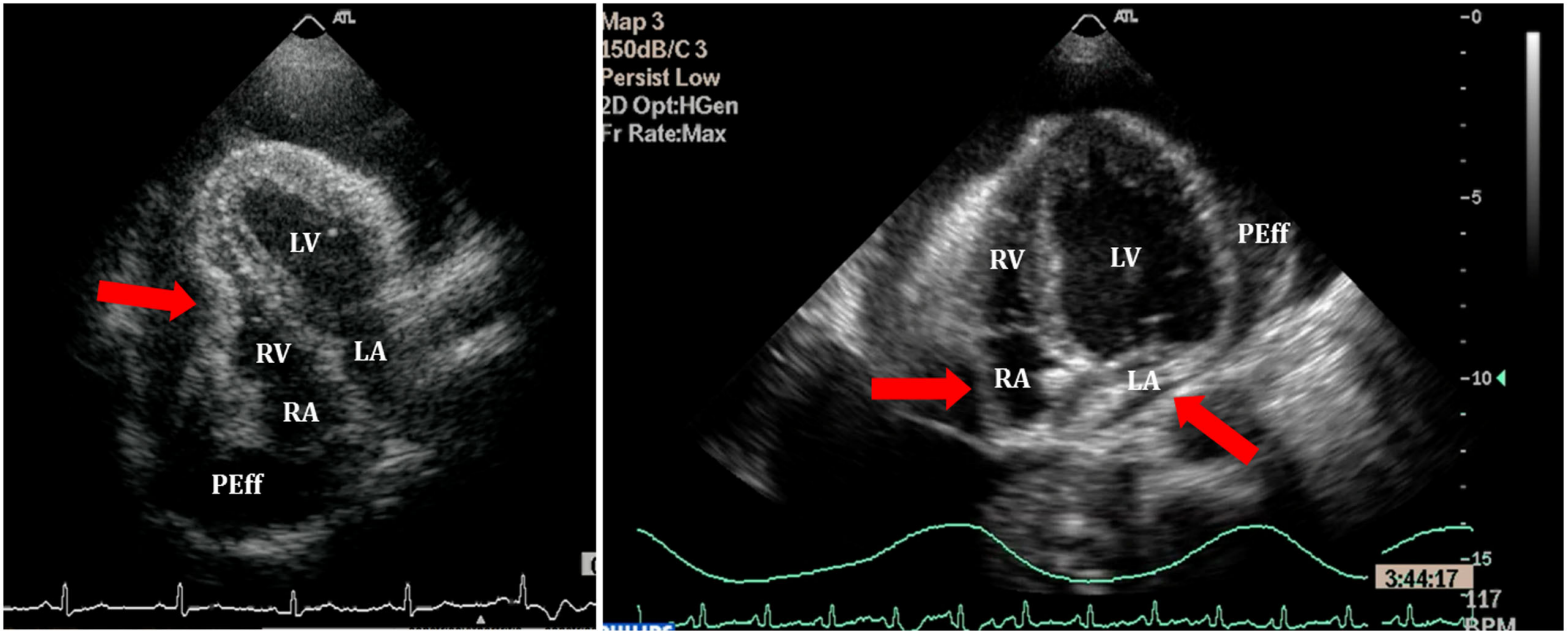
**Right ventricle (RV) collapse (left) in early diastole (end of T wave) in a patient with cardiac tamponade**. On the right, collapse of both atria in another patient with signs of tamponade.

**Figure 9:**
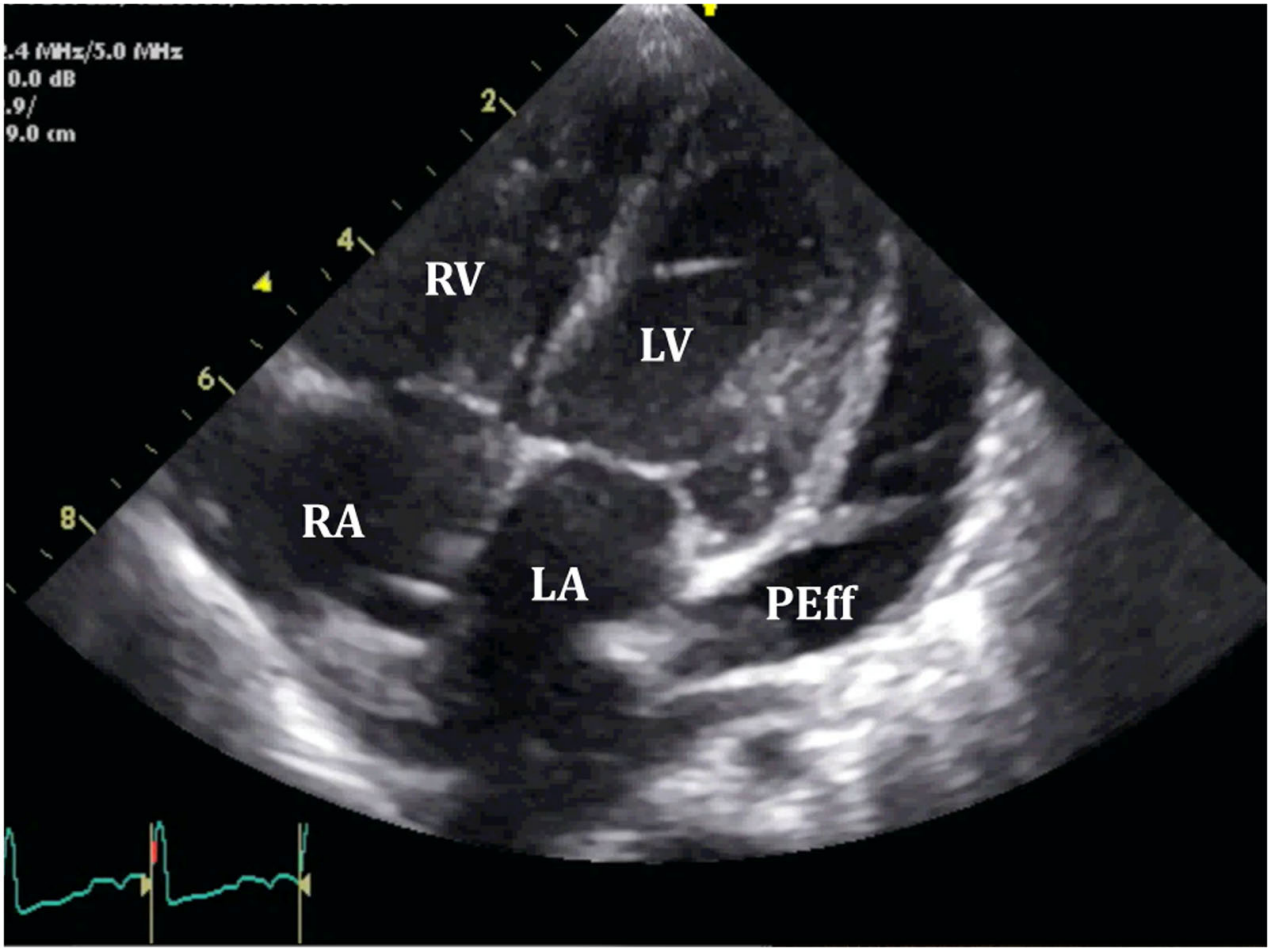
**Loculated severe post surgical pericardial effusion around the left ventricle (LV)**. Fibrin strains are also seen within the fluid.

**Figure 10:**
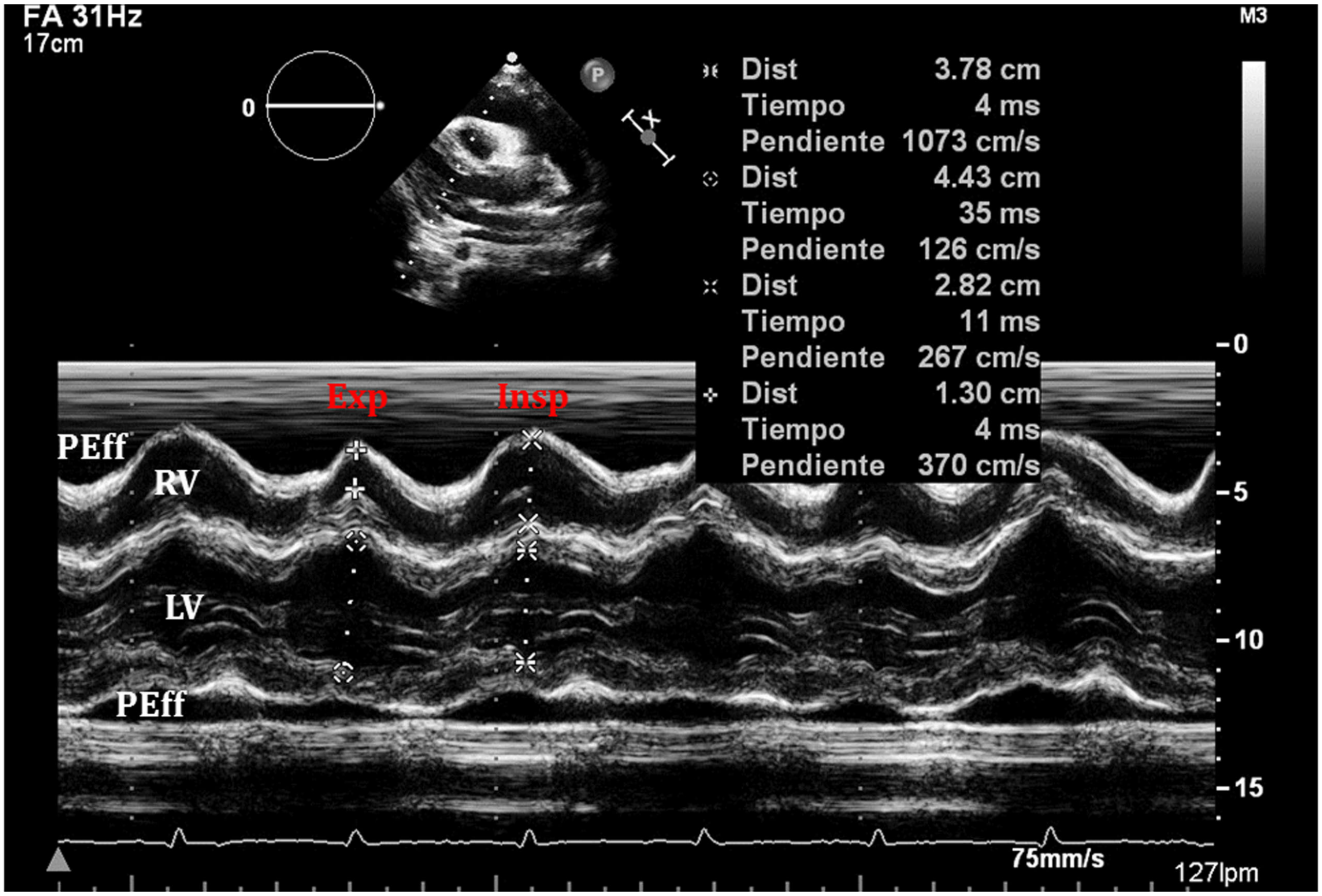
**An exaggerated ventricular interdependence following significant pericardial effusion shows increased right ventricle (RV) diastolic diameter during inspiration with decreased diameter of the left ventricle (LV), with the opposite changes happening on expiration**.

**Figure 11:**
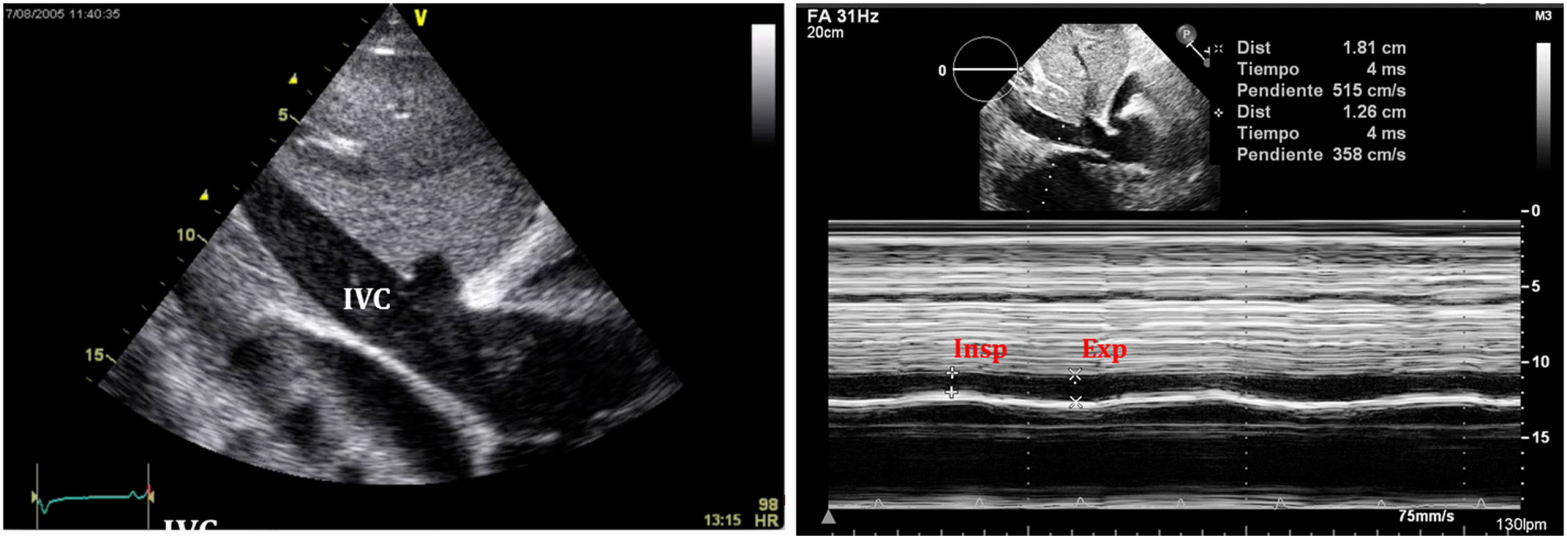
**Typical image of inferior vena cava (IVC) plethora (left) in a patient with significant pericardial effusion**. Collapse of IVC during inspiration is less than 50% (right).

**Figure 12:**
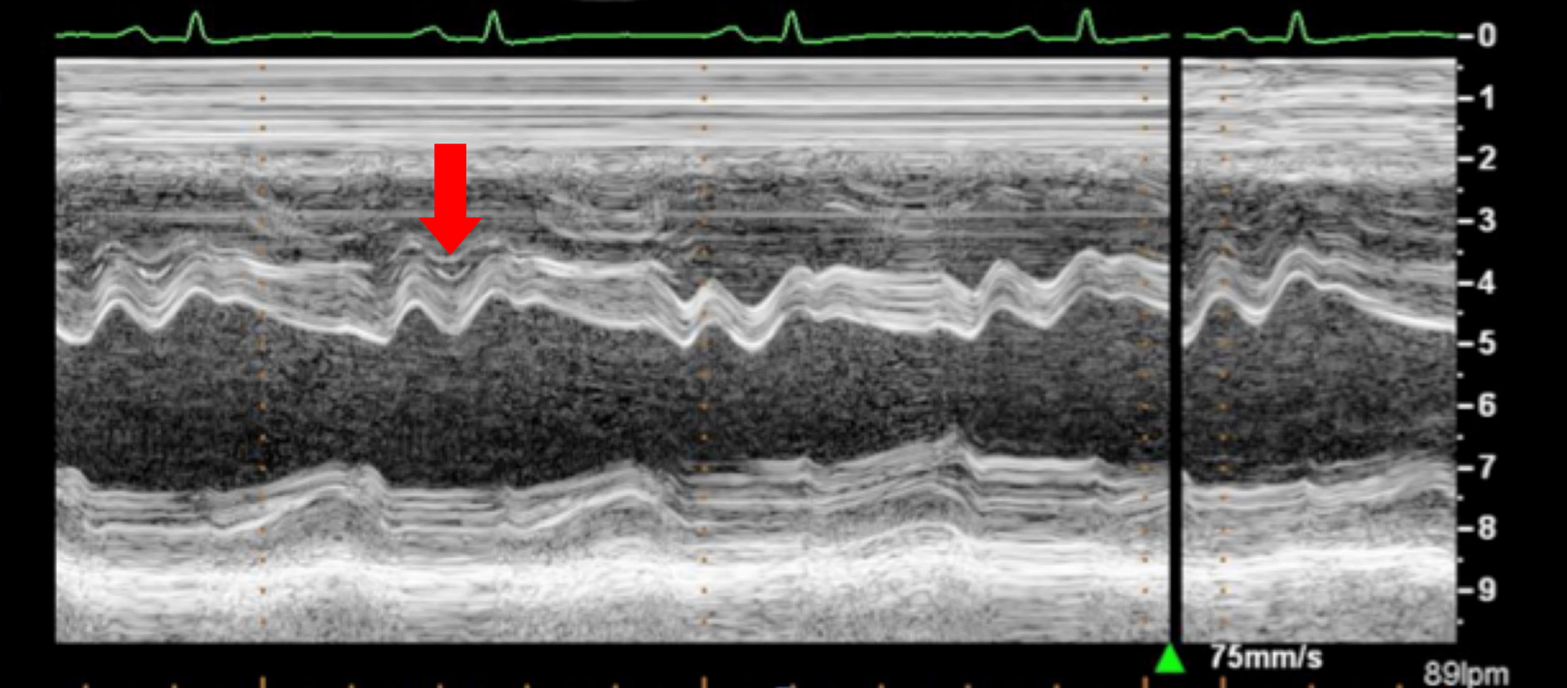
**Arrow shows paradoxical movement of the interventricular septum in early diastole suggesting elevated right ventricle filling pressures**.

(i) Quantitative and qualitative assessment of pericardial fluid
  Quantitative and qualitative assessment can be made following comments made earlier regarding PEff.
(ii) Collapse of cardiac chambers
  A common sign of cardiac tamponade with significant hemodynamic compromise is collapse of the right atrium (RA) and the RV. This happens during their relaxation phase, when intra-chamber pressures are lower than pericardial pressures (26). Atrial and ventricular collapses are observed at different points of the cardiac cycle. Atrial collapse is usually observed before ventricular collapse in the sequence of progressive cardiac tamponade.
  Right atrium collapse is commonly observed during systole. In early systole (near the peak of the R wave), intracavity pressure is lower and the atrial indentation of the thin free wall is seen. Moreover, duration of atrial collapse (collapse longer than one-third of the cardiac cycle) has been described as an almost 100% sensitive and specific sign of clinical cardiac tamponade (27) (Figure 7). While isolated RA collapse is frequently observed, collapse of the left atrium is, although described, rarely observed as a single chamber collapse (28). It is usually seen in cardiac tamponade along with collapse of the RA (Figure 8). Collapse of both atria increases the sensitivity and specificity of cardiac tamponade.
  Right ventricle collapse is commonly observed in diastole. During early diastole (at the end of the T wave), intracavity pressures are lower (Figure 8). Collapse of the free wall appears with moderate increases in pericardial pressures and will decrease stroke volume, initially without significant changes in the systemic blood pressure due to compensatory mechanisms (29). Initially, collapse of the RV free wall will only be present during expiration, but as collapse progresses, detection is possible throughout the respiratory cycle. Duration of collapse of the RV free wall is again an indicator of severity (30). Collapse will last as long as pericardial pressures remain higher than RV filling pressures. Thus, the longer the indentation on the free wall, the more severe the tamponade. M-mode through the affected wall (always along with appropriate EKG tracing) is useful to assess duration and timing of collapse. Experimental studies demonstrate that RV diastolic indentation is more sensitive, specific, and has a better predictive value of cardiac tamponade than *pulsus paradoxus* (26). Collapse of the LV is unusual, due to its thicker wall and may be seen in patients with severe pulmonary hypertension (31) or loculated PEff, typically around the free posterior wall of the LV following cardiac surgery (32) (Figure 9).
  All these echocardiographic signs of cardiac tamponade may not be present in those cases in which right ventricular diastolic pressures are elevated at baseline such as in pulmonary hypertension, positive pressure ventilation, severe LV failure, or other cardiac congenital or inherited conditions that would increase RV diastolic pressures (33, 34). On the other hand, collapse of the right chambers may occur earlier than expected in those conditions with reduced baseline intracavitary pressures, such as hypovolemia (35). Even with these limitations, absence of collapse of any cardiac chamber has a 90% negative predictive value (36).
(iii) Diastolic ventricular size variability with respiratory cycle
  When cardiac tamponade is present, M-mode in both parasternal long and short axes is a useful tool to assess exaggerated ventricular interdependence with the respiratory cycle. During inspiration, RV filling is increased, while LV size in diastole decreases (see Figure 10). The opposite scenario is present during expiration. This is a physiological phenomenon that can cause a variation of no more than 5% of the cardiac output in absence of tamponade (10).
(iv) Assessment of IVC
  An important sign of tamponade seen in 2D echocardiography is dilatation of the IVC (>20 mm in an adult size heart) and hepatic veins (Figure 11). This is known as IVC plethora, and, although not very specific, it is a very sensitive sign of cardiac tamponade (92%). A decrease of the physiological collapsibility of the IVC during inspiration is the commonly observed sign. M-mode through the IVC will demonstrate a less than a 50% reduction of caliber in most cases of significant PEff (Figure 11) (37).
(v) Septal “bounce”
  An inspiratory “bounce” of the interventricular septum toward the LV is a common, but not specific finding in cardiac tamponade (Figure 12). An M-mode through the parasternal long axis will show this abnormal movement of the interventricular septum. It may not be present in case of LV hypertrophy or increased LV filling pressures.

#### Doppler Echocardiography

Pulsed-wave Doppler is key in the assessment of respiratory variations of the transvalvular flows in patients with cardiac tamponade (32). In spontaneously breathing healthy individuals, Doppler peak velocities in the tricuspid and pulmonary valves increase during inspiration, while they decrease in the mitral and aortic valves. As previously mentioned in this review, a physiological variation of the cardiac output of around 5% exists with normal respiratory cycle, which is reflected in normal peak E-wave variation of up to 10% (7).

In cardiac tamponade, these variations in the Doppler velocities are larger.

**Figure 13:**
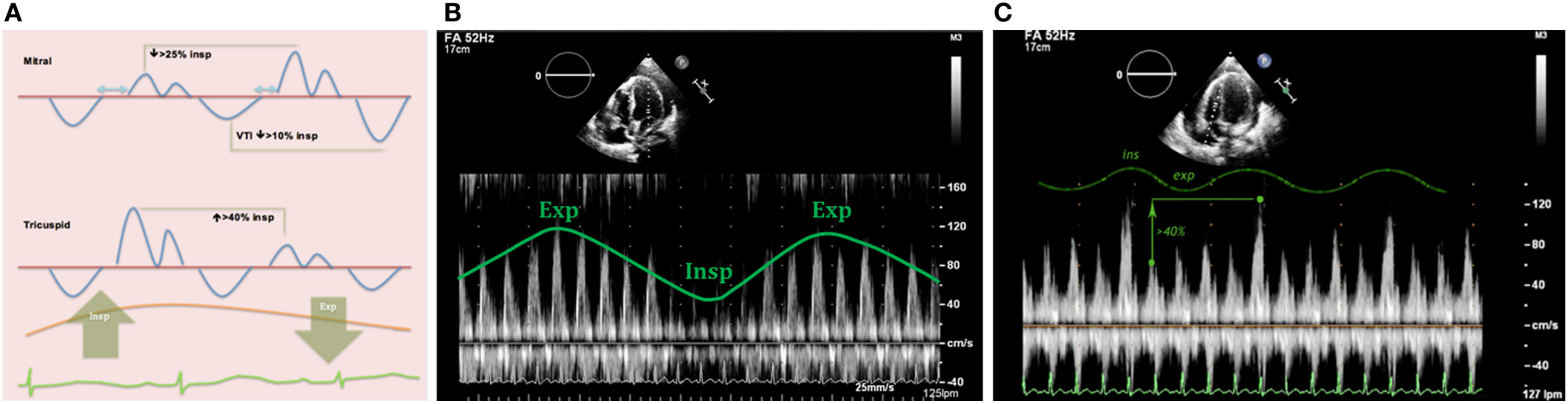
**(A)** Diagram showing mitral and tricuspid valves inflow patterns variability with the respiratory cycle in cardiac tamponade physiology. **(B)** Mitral peak E-wave of 119 cm/s (expiration) and 58 cm/s (inspiration), 48% drop in mitral E-wave velocity. **(C)** Doppler through tricuspid valve in the same patient shows an increase in peak E-wave (>40%) during inspiration.

**Figure 14:**
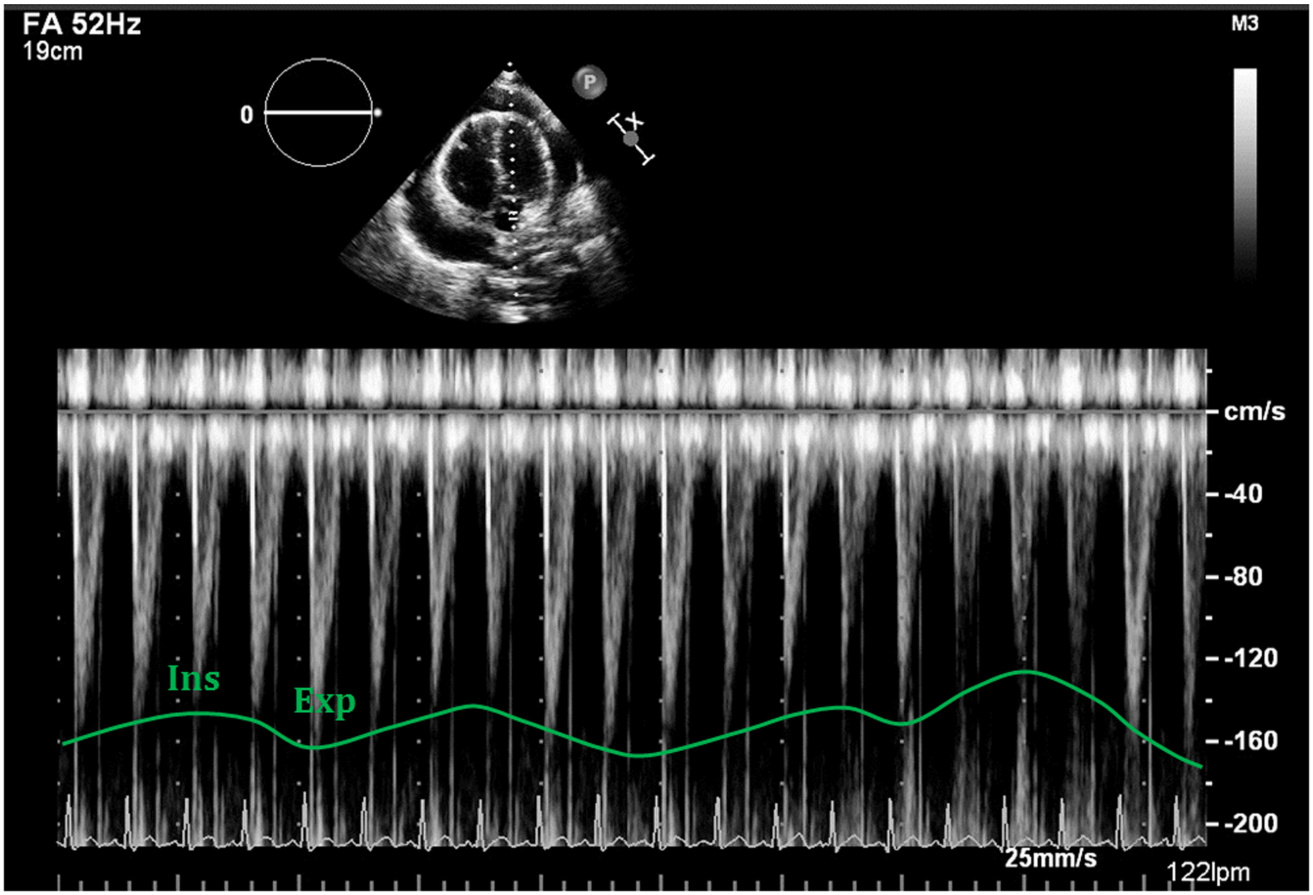
**Respiratory variability seen with Doppler analysis of the left ventricular outflow tract, demonstrating decrease of >10% following deep inspiration**.

**Figure 15:**
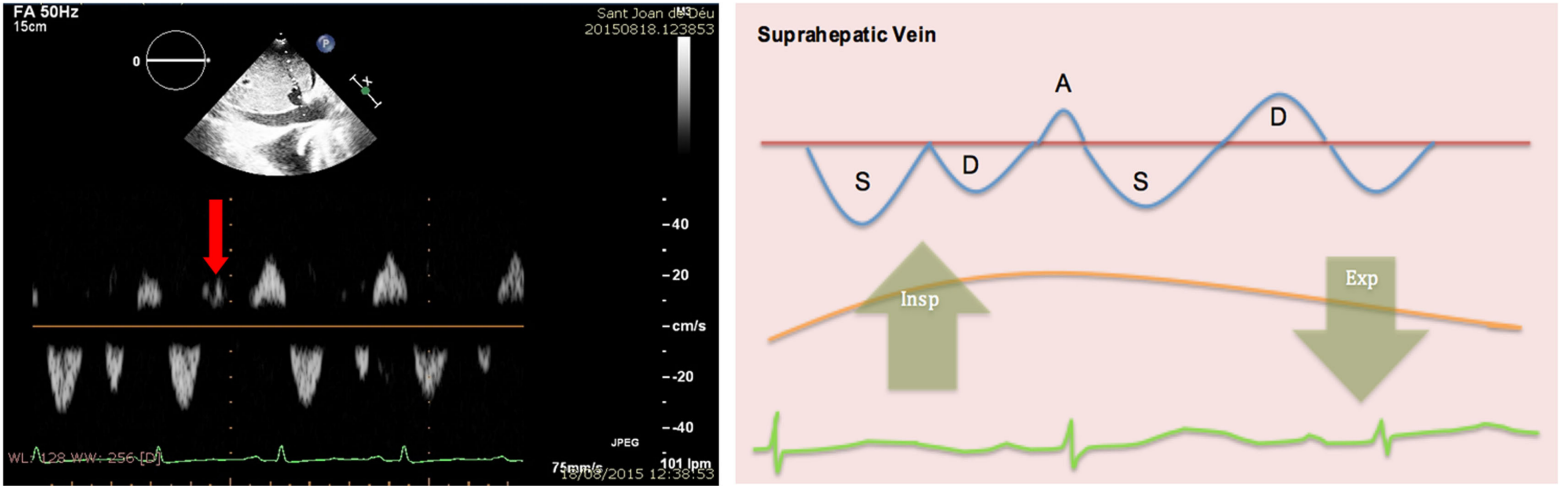
**Pulsed-wave Doppler through hepatic veins shows reversal of the normal D wave on expiration (left)**. Diagram showing changes of normal pattern in cardiac tamponade (right).

1. Mitral valve: research studies demonstrate an inspiratory reduction in mitral peak E-wave velocity in cardiac tamponade of at least 25% (32, 33, 36). The value of the E-wave peak will vary based on the phase of the respiratory cycle at the time of early diastole (lower filling pressures) (Figure 13).
2. Tricuspid valve: peak E-wave tricuspid valve physiological variation is larger than the mitral valve fluctuations. In cardiac tamponade, the peak E-wave velocity will drop at least 40% in expiration compared to inspiration (changes seen mainly in first two heart beats of expiration) (Figure 13).
3. Right and left ventricular outflow tracts: physiological variations in aorta and pulmonary trunk are typically less than 10%. In presence of hemodynamically significant PEff, peak velocities in both outflow tracts will show a larger difference with respiratory cycle. During inspiration a drop of >10% of the peak velocity will be seen in the aorta (Figure 14), while the opposite will happen in the right ventricular outflow tract, where an increase of at least 10% will be noticed (38).
4. Hepatic veins: normal flow pattern shows a biphasic wave followed by a reversal wave corresponding with atrial contraction (see Figure 15). In cardiac tamponade, while venous return will still increase with inspiration, decreased or even reversal flow will be seen in diastole (before atrial contraction). High positive and negative predictive values have been reported for changes in Doppler signal in hepatic veins (82 and 88%, respectively) (36). Nevertheless, this has some limitations since hepatic vein flow is difficult to assess in up to one-third of patients.
5. Pulmonary veins: pulmonary vein flow shows a biphasic wave with reversal flow during atrial contraction. During inspiration, D wave is smaller due to reduced left ventricular filling (increased LV filling pressures). In expiration, S wave is larger following decrease in LV filling pressures (increased flow toward the ventricle).

### The Verdict

Echocardiography is the technique of choice for the assessment of PEff. Nevertheless, as stated in this review, published data continue to reveal a large variability in specificity and sensitivity of the typical echocardiographic findings in patients with significant PEff. Of all assessments, the absence of collapse of any of the cardiac chambers seems to be the echocardiographic sign with a higher and more consistent negative predictive value to rule out cardiac tamponade. Unfortunately, although very helpful, none of these echocardiographic findings is 100% diagnostic of this critical condition. Thus, final decision and management of PEff should always be made following a detailed physical examination and a proper clinical assessment.

## Ultrasound-Guided Pericardiocentesis

When clinical findings and echocardiography signs of cardiac tamponade are present, pericardiocentesis must be performed without delay. Ultrasound is useful before, during and after the procedure. Its use has shown to reduce the incidence of major complications, reported as 3%, comparing to the blind subxiphoid approach that has an associated incidence of complications of 5–25% (39–41). Assessment of the effusion must be done from standard views to find the largest area of effusion closest to the transducer avoiding vital structures. Several approaches have been used, and a long-axis in-plane-guided insertion using a linear probe has been recently validated as an easy and safe method to perform the pericardiocentesis in small children (42). Access from the anterior chest wall, typically subcostal, is the most common point of incision. Parasternal left access (between fifth and sixth intercostal spaces) can also be used, but that carries a higher risk of pneumothorax. With either approach, Seldinger technique is typically used and placement of a pigtail drain is usually recommended. During the whole procedure, correct positioning of the wire and the catheter should be monitored with echocardiography (43).

## Author Contributions

A-PC: primary author, writing the bulk of the manuscript; image acquisition. L-BG: image acquisition and image preparation, writing sections. S-C: image acquisition and image edition, critical revision of the manuscript, and writing sections. J-SdT: drafting of the manuscript, image acquisition, editing, critical revision of the manuscript, and responsible for the overall content.

## Conflict of Interest Statement

The authors declare that the research was conducted in the absence of any commercial or financial relationships that could be construed as a potential conflict of interest.

## Abbreviations

LV, left ventricle; RV, right ventricle; LA, left atrium; RA, right atrium; CMR, cardiovascular magnetic resonance; CT, computed tomography; PEff, pericardial effusion; IVC, inferior vena cava.
